# An in-depth qualitative study of health care providers’ experiences of performance-based financing program as a nation-wide adopted policy in Cameroon: A principal-agent perspective

**DOI:** 10.1371/journal.pone.0288767

**Published:** 2023-07-28

**Authors:** Miriam Nkangu, Julian Little, Raywat Deonandan, Roland Pongou, Sanni Yaya

**Affiliations:** 1 School of Epidemiology and Public Health, University of Ottawa, Ottawa, Canada; 2 Health Promotion Alliance Cameroon (HPAC), Yaounde, Cameroon; 3 Interdisciplinary School of Health Sciences, University of Ottawa, Ottawa, Canada; 4 Department of Economics, University of Ottawa, Ottawa, Canada; 5 School of International Development and Global Studies, University of Ottawa, Ottawa, Canada; 6 The George Institute for Global Health, Imperial College London, London, United Kingdom; University of Zambia Institute of Economic and Social Research, ZAMBIA

## Abstract

**Objectives:**

The study applies the principal-agent approach to explore providers’ experiences before and after the introduction of performance-based financing (PBF) in Cameroon, challenges and facilitators in the implementation process, and mechanisms in place to ensure sustainability.

**Methods:**

The study was an in-depth qualitative study whose goal was to provide multiple descriptions of experiences and insights from a principal-agent analysis perspective. Purposive sampling was used to identify the key characteristics of the participants relevant to the study. A snowballing technique was used to further identify eligible participants. Only healthcare providers who were exposed to the previous system and could reflect on and provide meaningful data that captured the everyday experiences before and after the implementation of PBF were included. Data were collected from three districts in the Southwest region of Cameroon from May 2021 to August 2021. Data were transcribed and analyzed using MaxQDA.

**Results:**

A total of 17 interviews and 3 focus group discussions (24 participants) were conducted with healthcare providers and key stakeholders involved in PBF. The respondents described a range of changes that they had experienced since the introduction of PBF. Each of these changes was categorized as either positive or negative. Positive changes were framed into 14 dominant categories: motivation, negotiations, innovation, resource allocation, autonomy, decentralization, transparency, improved quality of care, separation of function, performance, equity considerations, opportunity to recruit, participation in decision-making, and improved access to and utilization of maternal health services. The main challenges (negative experiences) reported were framed into nine categories: management of change, retention issues, conflict of interest, poor understanding of the PBF concept, resistance to change, verification challenges, delays in payment of PBF incentives, data entry and documentation, and challenges in meeting the equity considerations of the poor and vulnerable. Despite the challenges, providers preferred the decentralized approach to the centralized system.

**Conclusion:**

PBF is a national strategy for achieving universal health coverage in Cameroon, and the experiences of providers provide a vital guide to refine national policy. The introduction of PBF has provided positive changes to providers’ quality of care when compared to the previous system. Addressing the delays in PBF payments will help to overcome the challenges to implementation and provide opportunities for health facilities to be more efficient and improve their performance. Despite the limitations of delay in payment, PBF helps to align the incentives of the health workers (agent) with those of the Ministry of Health (principal).

## Introduction

Performance-based financing (PBF) is a supply-side strategy, with the primary objective of improving healthcare quality, boosting motivation, and improving the performance of health workers [1–12]. Therefore, the health provider is the key client (agent). The principal-agent (PA) theory [1,12] has been used to explain PBF within the health sector, where the interest of the principal (in this context, the Ministry of Public Health) does not necessarily align with that of the agent (the health worker) [1,12]. Given the centralized nature of health systems, the interests of the principal and the agent are independent and there is usually information asymmetry between the two parties [1,12]. Some actors have perceived PBF as an ongoing process of decentralization that involves tensions and continuous negotiations [13]. Thus, evidence generated in relation to its effectiveness is likely to vary by time and space [13].

The introduction of PBF has led to drastic changes in the daily activities of healthcare providers in relation to documentation procedures, verification processes, quality checklists, participation, and autonomy [1,12,14]. Following the adoption of PBF as a national strategy in Cameroon in 2017, health facilities have been expected to sign PBF contracts on a quarterly basis. These contracts describe the conditions required to obtain PBF subsidies. Based on information gathered from the PBF team, the requirements include efforts to improve management, minimum quality levels, governance and financial accountability and clauses for termination of the contract. There are verifications and sanctions, with detailed rules for quality. Health facilities are expected to present business plans on a quarterly basis; and if they are unable to meet this expectation, it is the responsibility of the regulatory body to intervene and ensure that measures are put in place to address it. In addition, it is not sufficient for the health facility to present its business plan; these plans should also adhere to the conditions specified in the contract. For example, for routine renewal of a contract, a minimum quality assessment score of above 70% is needed. However, for newly enrolled health facilities, there is a grace period of approximately three quarters, with the expectation that the health facility will meet the minimum quality standard in subsequent quarters. After such time, if a quality assessment is below 50%, the contract is unlikely to be renewed. Such facilities are then expected to reinforce their strategies to raise the quality to the minimum before the contract can be renewed; otherwise, they are sanctioned by the regulatory body.

Research on the experiences and opinions of service providers in the context of PBF is scarce, despite the policy aiming to boost motivation and improve performance. The experiences and challenges of healthcare providers and PBF implementers are central to the implementation, acceptability, and potential for sustainability of the policy, especially in Cameroon, where the policy has been adopted nation-wide.

As PBF is a donor-driven policy, local governments struggle to sustain the policy after implementation [15–17]. For example, in West Africa, some countries—such as Benin, Sierra Leone and Mali—initially implemented PBF and then discontinued the programs after the withdrawal of donor funding [6–8]. Countries such as Cameroon, Rwanda, and Burundi have reached scaling at a national level with domestic support [9]. However, in Cameroon, the aspects of implementation and providers’ experiences, given that it is a new policy aimed at improving the quality and performance of health providers, remain understudied.

Health providers in Cameroon and across sub-Saharan Africa have long been inadequately paid and poorly motivated [2]. This—among other factors—has led to poor performance, staff absenteeism, substantial out-migration, and rural-urban migration, resulting in a great financial loss to the economy and weakening of the health systems [2]. PBF is seen as a powerful mechanism to boost staff motivation and improve quality and performance [10,11]. This paper builds on the first author’s assessment in 2008 of health workers’ performance and quality of care delivery across sectors in rural and urban settings in Cameroon at a time when Cameroon’s health system was centralized and PBF had not yet been implemented [18]. Most healthcare providers (from public and confessional sectors) reported low motivation, staff absenteeism, poor performance, and dissatisfaction with the health system and were looking for opportunities to either supplement their income or migrate to find better working conditions [18]. A decade later, Cameroon adopted PBF to strengthen its health system, improve quality of care, and address some of the issues faced by health workers. It is important to explore the experiences and challenges of health providers, before and after PBF, and the impact on quality of care and performance of health providers.

The present paper reports an in-depth qualitative study that aimed to provide multiple descriptions of experiences and provides some insights from a PA analysis perspective. We carried out in-depth interviews to explore health professionals’ experiences and allow them to reflect on their experiences and changes to the previous structures and the newly implemented PBF approach, thus enabling previously silent voices to be heard. We investigated providers’ experiences before and after PBF, and the challenges and facilitators in the implementation process. We examined the impact of PBF on health professionals’ day-to-day activities in the process of care delivery. We also sought to identify the challenges in the implementation that could hinder the sustainability of the policy, and what mechanisms in place could be reinforced to ensure sustainability.

## Methods

### Context and settings

Cameroon is a lower-middle income country (LMIC) in sub-Saharan Africa, with a population of approximately 26 million as of 2020 [19]. Health care is delivered in the private, public, and para-public sectors. The private sector is composed of for-profit and confessional health facilities. Based on a 2012 Ministry of Health assessment, the country has 1,888 publicly integrated health centers, 760 private health centers, 155 sub-divisional health centers, and 164 district hospitals [5]. The Ministry of Health defines the health policy, the regional level translates the health policy for operations, and the district level operationalizes the health policy into action. The proportion of GDP spent on healthcare was 3.6% in 2019 [19–21] and the operational level receives a small fraction of this health budget [22]. Most resources are allocated to administration and infrastructure, rather than the operational level, where all activities are implemented [22].

There are ten administrative regions in Cameroon, two of which are English speaking, namely the Northwest and the Southwest. There has been armed conflict in both English-speaking regions since 2017, but the Southwest region was relatively accessible at the time of the present study. Health districts from the Southwest region were included in the PBF pilot study in 2012 and the PBF impact evaluation assessment of 2017 [22]. We carried out this study in the Buea, Limbe, and Tiko districts within the Southwest region.

### Study design

This study uses a qualitative research approach. In depth interviews offer an opportunity to capture rich and detailed information about respondent’s points of view, experiences, feelings, and perspectives before and after the implementation of PBF [23]. Given the centralized nature of Cameroon’s health system before PBF, the study employs the PA lens, which is documented among the essential issues underlying the health systems in most sub-Saharan African countries [1,7,9–11], where the interests of the principal and agent are not necessarily aligned, given the centralized nature of health systems. Thus, PBF was viewed as a powerful tool to decentralize health systems, in principle addressing the PA problem [7,7–11]. This study employs the PA approach to understand the experiences and challenges of healthcare providers transitioning from a centralized to a decentralized approach in their day-to-day practice.

### Sampling and participant recruitment

A sampling frame was used to group the health facilities in each of the three districts by sector. The sample consisted of PBF managers from the national and regional levels, healthcare providers from across sectors, and district medical officers and the regional representatives in this context are the regulators. This was done to gain meaningful data on their similarities and variations. The sample size was determined by the depth of the data and the emergence of common themes, with a saturation point ultimately reached, at which point no new information was reported [23]. Purposive sampling was used to select participants based on their role and the time during which they had worked in that role. Thus, we defined and identified potential eligible participants as health providers or administrators who had been working with the health system (at each health facility) before the implementation of PBF in the health facility. A snowballing technique was used to further identify eligible participants. The rationale for selecting participants within this time range was that PBF was implemented in this region from 2012 in a small number of facilities (in Buea and Limbe) and only later, in 2018, it was scaled in Tiko district and across all health facilities. Thus, only healthcare providers who were exposed to the previous system could reflect on and provide meaningful data that captured the everyday experiences before and after the implementation of PBF. In this study, the timing (before and after) was based on when the health facility enrolled into PBF. Health providers hired at the health facility when PBF was already in effect at the facility were not eligible. In addition, the breadth and depth of their experience over time had enhanced their understanding of the health system, while their exposure to PBF (over a minimum of 4 years) provided valuable insights into potential sustainability mechanisms and approaches that could inform the PBF program in Cameroon. To minimize recall bias, we compared the responses of health care providers from the health facilities that enrolled into PBF in 2012 versus facilities that enrolled in 2018.

### Data collection

The first draft of the interview guide was developed by the primary investigator and revised by the co-authors. The interview guide comprised six key questions in relation to the health professionals’ experiences, perceptions of challenges, and considerations of potential for sustainability, with flexibility for probing as appropriate. The interviews commenced with warm-up questions that concerned the general overview of the discussion and provided a more comfortable environment for the participants. Warm-up questions were asked about PBF and the interviewee’s role in the program or health facility. These were followed by specific questions and probes about their experiences with the newly implemented reform, as compared to the previous system of operation; their understanding of the PBF policy; the challenges and facilitators; and the policy’s potential for sustainability. Each interview ended with an opportunity for the interviewee to propose their own policy recommendations and suggest areas to be addressed. The same questions were put to administrators and PBF managers, with only slight changes to reflect the participants’ respective roles. The data were collected between April 2021 and August 2021.

An in-depth semi-structured interview format was used to allow participants to freely express themselves. All the interviews were conducted by the researcher (MN) and recorded. The interviews were primarily conducted face-to-face in the offices of the interviewees. The interviews were audio recorded and varied in length, ranging from 30 to 45 minutes each. No relationships were established between the interviewer and a participant before the start of the interview. The purpose of the study was explained to the participants before the interviews, and informed consent was obtained. Field notes were collected during the interviews to record relevant non-verbal considerations or actions—for example, the tone, mood, and coherence of the respondents. Each participant’s data were coded to ensure confidentiality and anonymity. Three focus groups were conducted with community health workers (CHWs) at a convenient location, with one group for each district, and each lasted for up to 1.5 hours. We obtained written informed consent from all participants. Participants were also informed that once they chose to participate, they could withdraw at any time or choose not to answer any questions, for which there would be no negative consequences.

### Data processing and analysis

After the interviews, the audio-taped recordings were transcribed verbatim and compared with the recordings for accuracy. The transcription was done with the help of research assistants. The data were read several times, allowing the researcher to become immersed and to make notes and reflections. The data were exported into MaxQDA software v2020 [24], and the initial codes were developed using the participants’ own words and the field notes, giving specific attention to statements that reflected their experiences and understanding, the challenges and facilitators, and sustainability. The lead author (MN) and a research assistant conducted the initial coding, then the initial codes were compared for consistency and to ensure the trustworthiness of the process, with attention given to experiences of the implementation processes, the challenges, and the individual’s understanding of the PBF policy. Keywords and phrases were documented by the investigator and research assistant, as they engaged with the data. The notes were grouped into a coding scheme to create subcategories. These subcategories were then compared, contrasted, and revised by co-authors SY and JL. At a later stage, subcategories reflecting similar content, trends, and understandings were merged to produce main themes. Two independent coders (MN and a research assistant) independently analyzed the transcript and coded the data, using various themes to assess the inter-coder reliability and trustworthiness of the findings. Inter-coder reliability was discussed among the coders, who shared varied interpretations to enrich and finetune the analysis and, ultimately, to converge into a shared interpretation of the data. To ensure confidentiality, no personal information was included in transcripts and quoted texts. Member checking was conducted with key respondents to ensure the interpretation of the data reflected their responses and no major changes were made. The findings are reported using the COREQ criteria for qualitative interviews [25].

### Ethical considerations

The Bruyere Research Institute at the University of Ottawa approved the study (# M16-18-057), and administrative clearance was obtained from the Faculty of Science, University of Ottawa (#H-02-19-2829). In Cameroon, we obtained ethical approval from the University of Buea Faculty of Health Sciences (Ref 2020/1342-02/UB/SG/IRB/FHS) and administrative clearance from the regional delegation of public health in the Southwest region (Ref R11 MINSANTE/SWR/RDPH/PS864/715).

## Results

### Characteristics of participants

A total of 17 interviews were conducted with key stakeholders involved in PBF: the national and regional technical PBF unit (3); Ministry of Health regulators (3); health providers, including nurses, midwives, and doctors (11) (see Table 1); and three focus group discussions with CHW, one FGD per district (24 participants) (see Table 2). The providers were from private, public, and confessional facilities. Facilities were enrolled in PBF at two time points, 2012 and 2018. The responses were similar across all facilities and districts.

**Table 1:** Characteristics of health providers and administrators for key informant interviews.

Participants	Tiko	Limbe	Buea
Health care providers			
Nurses	2	1	2
Midwifes	1	0	2
Doctors	2	1	0
Administrators including regulators and PBF managers (referred in text as stakeholders)	2	1	3
**Total**	**7**	**3**	**7**

**Table 2:** Socio-demographic characteristics of respondents for focus group discussions for CHW in Buea, Tiko, and Limbe districts.

Participants	Age	Level of education	Marital status	Employment status	District
**CHW1 Male**	39	High School	Married	Employed	Buea
**CHW2 Male**	28	High School	Single	Employed	Buea
**CHW3 Woman**	38	-	Married	Not working	Buea
**CHW4 Woman**	39	Primary	Married	Employed	Buea
**CHW5 Woman**	27	-	-	Not working	Buea
**CHW6 Woman**	34	Secondary	Single	Employed	Buea
**CHW7 Woman**	32	Secondary	-	Not working	Buea
**Participants**					
**CHW1Male**	33	University	Married	Employed	Tiko
**CHW2Male**	41	High School	Married	Employed	Tiko
**CHW3 Woman**	42	High School	Married	Farming	Tiko
**CHW4 Woman**	43	Primary	Married	Employed	Tiko
**CHW5 Woman**	39	Secondary	-	Employed	Tiko
**CHW6 Woman**	33	Secondary	Married	Not working	Tiko
**CHW7 Woman**	29	Secondary	-	Employed	Tiko
**CHW8 Woman**	40	Secondary	Single	Not working	Tiko
**CHW9 Woman**	26	University	-	Student	Tiko
**CHW10 Woman**	28	Primary	Single	Not working	Tiko
**Participants**					
**CHW1 Male**	28	Secondary	Married	Employed	Limbe
**CHW2 Male**	43	Secondary	Single	Self employed	Limbe
**CHW3 Male**	49	Primary	Married	Employed	Limbe
**CHW4 Woman**	28	Secondary	Married	Not working	Limbe
**CHW5 Woman**	38	Secondary	Single	Employed	Limbe
**CHW6 Woman**	42	Secondary	Single	Farming	Limbe
**CHW7 Woman**	47	Secondary	Single	Farming	Limbe

Not working implies they do not have a contract with a health facility under the PBF program.

The results were categorized into four primary themes, with subthemes that reflected the positive and negative experiences: The respondents described a range of changes that they had experienced since the introduction of PBF. Each of these changes was categorized as either positive or negative. Positive changes were framed into 14 dominant categories: motivation, negotiations, innovation, resource allocation, autonomy, decentralization, transparency, improved quality of care, separation of function, performance, equity considerations, opportunity to recruit, participation in decision-making, and improved access to and utilization of maternal health services (see Table 3). The main challenges (negative experiences) reported were framed as nine categories: management of change, retention issues, conflict of interest, poor understanding of the PBF concept, resistance to change, verification challenges, delays in payment of PBF incentives, data entry and documentation, and challenges in meeting the equity considerations of the poor and vulnerable.

**Table 3:** Themes from data.

Themes	Positive categories	Negative categories
**1**.Transition from centralized to decentralized system	Motivation, autonomy, decentralization, negotiations, opportunity to recruit, transparency, separation of functions, participation in decision making	Conflict of interest, resistance to change
**2**.Quality of care	Performance, process of care, innovative, equipment, motivation	Proper documentation, data entry, verification challenges
**3**.Access to care	Home visits, equity considerations, resource allocation, subsided cost	
**4** Delays in payment		Meeting equity consideration, verifications, retention, conflict, performance

### Transition process from a centralized to a decentralized system

The process of change was characterized by enormous challenges at all levels. These challenges were not unexpected, as this was a new reform running parallel to the existing health system and involving a process of decentralizing a centralized system. The data revealed that these challenges persisted while the system underwent the process of adaptation. The changes experienced at the national level were accompanied by conflicts of interest, resistance, and sometimes threats, especially in relation to maintaining the status quo and an attempt to reverse the allocation of financial resources. Drawing from a PA theory analysis, these tensions are obvious and enables the process of negotiations and readaptation of strategies.

*“As a new concept the key challenges was how to adequately adapt the principles within existing laws, rules, regulation, habit. Habits that have been developed over time that are even more than laws. To get those who are supposed to implement these PBF principles to adhere correctly to the principles by shifting away from their old habits, is a big challenge. Thus, they are not only challenges but also threats…… we are very conscious of that, that is why there is that component of constant sensitization, constant coaching, supervision, and re-adaption of strategies.” (Interview: Stakeholder)*.

*“PBF is a reform coming in another context that have laws in place for example, Cameroon is a centralized system, and you want to decentralize decisions, it causes friction between PBF and the system. For example, in PBF, you have to manage participation of all personnel, and transparent, it is not usual with the directors in a centralized system to sit with their personnel and decide on how to spend the budget and take decisions, they want to decide alone and send the decisions to the personnel and that is not something easy to break through” (Interview: Stakeholder)*

Participants explained the importance of aligning PBF to the national context, as well as adapting the principles of PBF to the realities of the country, because a policy cannot simply be transferred from one context to another. The key issues were around linking performance to financing and shifting away from financing inputs and processes that do not yield results to a process of buying results. One major challenge in the process of change was related to the limited budget that flowed to the operational level before PBF was introduced. As noted earlier, this limited budget flow had affected the daily functioning of the operational level. However, with the adoption of PBF, some major process of reversing this budget allocation has been met with resistance.

*“One of the issues is also that the operational level needs more money than the policy making level, so they needed to reverse the policy, and this was also a challenge in breaking through this process. This, however, has been taken care of and we have already changed the policy only the Ministry of Finance pays in cash now to health unit, and they paid based on performance not just as they use to give and you decide how you use it, that is the key policy change we have been able to achieve, with a lot of resistance” (Stakeholder)*

*Before the coming of PBF, source of funding all came from credit cards, motivations like mission orders, fuel order. But now, the coming of PBF has opened the eyes of every stakeholder concerned, that if you work, you benefit, if you don’t work you don’t benefit. It has come to make us understand our job description. For example, if I’m called upon to give the financial management data, the database, the dashboard of finances on personnel, I should be able to give it, if I don’t give they evaluate me and [I get] no benefit”. (Interview: Stakeholder,Buea)*

The transition from a centralized to a decentralized system facilitated decision-making and participation among providers and promoted budget redistribution and improved motivation. An interesting observation was reported amongst providers regarding workload. While some providers argued that PBF has increased their workload in relation to documentation, others viewed the increased workload as part of their duties which they ignored in the previous system.

*“Now, documentation has increased, we are like no longer nurses, everything must be written, the registers keep increasing day in and day out. Today they bring this one tomorrow they bring another, so now you carry out a procedure you can enter that same procedure in about five different registers. So, it is like we are now secretaries and not nurses” (Interview: HCW, Limbe/Buea)*

*“Yes, documentation is another challenge with the coming of PBF, but I will not really say it is a challenge because those are activities we were supposed to carry out but because PBF was not in place, we usually ignored those activities for example, before PBF, I can consult a patient and don’t enter that patient details in my register and nobody will come and investigate me, but knowing fully well that there is this PBF scheme, they will come and validate and pay for my services, I have to register those patients so it has increased our workload when it comes to documentation, for example there is a form here for validation, the whole of this table is filled with registers upon registers, but I think that is just the right thing to do but we ignored those activities in the past before PBF” (Interview:HCW, Buea,Tiko,Limbe)*

*“Actually, PBF has come to boost our motivation because on like the previous years before 2012, some health district administrators had so many lapses. So, there is what we called, motivation of personnel, and motivation of personnel doesn’t only relate to finance, also job satisfaction, but PBF has come to make you to know that you will be motivated if you work, and it has increased our performance (Interview, HCW, Buea,Tiko,Limbe)*

### Quality of care

The providers reported positive changes in relation to quality of care, primarily in relation to performance, motivation, facility presentation, equipment, and the process of care. Most providers reported that PBF had helped the community that they served and had improved their own performance and quality of care delivery.

*“The services have changed compared to before. With PBF, we are working with standard quality, before PBF, we were just working. Also, education wise, PBF has help in improving knowledge like many things we did not see important what we neglected in healthcare practice before, PBF has made us understand that what we neglected before is actually important and can be paid more for that service. PBF has made us go extra miles to learn more skills and better manage such cases and produce quality” (HCW)*

*“The PBF system is excellent, in the previous system, we were just working vaguely, nothing defined, it has increased the level of consciousness of health personnel, patient data, statistics, increased small growing facilities to a mighty facility. We started as a small clinic to this structure and the other structure, if we were depended on the minimal profit we got, we would not have expended, but thanks to PBF we were able to increase and expand our services, in terms of equipment, infrastructure, inflow of patient and the motivation which facilitates the work” (Interview, HCW,Buea)*

*“I have been working before PBF was introduced and the difference is,.before PBF, some nurses usually talk to patients rudely, but now with PBF all this is gradually changing because when they realize you speak rudely to patients your name is being removed under the PBF” (FGD: CHW, Limbe,Buea & Tiko).*

*“Even the patients can also attest that the quality of services is better than before. Now we pay more attention to our patients, we make sure we check their vital signs when it is necessary, when we check we chart it because our files are being monitored. Before PBF. you could even imagine things in your mind and think that you can just carry on your functions without planning for them. But with the PBF, we are compelled to do it. If you don’t do it, you lose marks, and we know that you are paid for the services rendered. It is competitive so it gives people the enthusiasm and motivation to give their best. I believe that the difference is so clear from the time we enrolled into PBF and before. (Interview, HCW, Buea/Limbe)*

### Access to care

Most respondents reported an observed increase in access to care within their health facilities. This observed increase in utilization was also attributed to the introduction of equity aspects—which seemed to be a novel approach for providers—and to home visits. Home visits allow CHWs to reach out to poor and vulnerable individuals, especially those who may not have access to the system due to barriers of cost, social status, information, and vulnerability. The use of CHWs for home visits was reported by providers to have had a positive effect on access.

*“Home visits for PBF have really improved access. We often see situations that are deplorable, that invokes the feeling of pity, for example, there was a woman that was poor and vulnerable. She had three beautiful boys, but these children had terrible wounds which were in dangerous areas. I do not know if it was due to rough play or not, but we told her she could get treated for free and so she came and we treated them continuously, till the wounds were healed and even now when the children are sick, she comes to the facility and can even pay at times. So, the home visits are really important for it is there that we see those faced with challenges even kids who are malnourished and we are able to help. I really appreciate PBF for this” (Interview/FGD, HCW Limbe)*

*“I will say without PBF it would have been very challenging, without PBF I don’t think we would have had a building behind this consulting room. When the government gave us this structure, they told us our building will be over there…. They would have been telling us to wait… However, the coming of PBF has helped us to do a lot of things that normally we will not have been able because we will have still been depending on the government every time, we need something. But now, that is not the case. It has really helped us to expand, unlike before, this place used to be in cubicles, the hall was tiny, but now the hall is big. Before, when you were working, you could not have something substantial that you can invest. We were just working and consuming, now when we work PBF has a percentage, and we have a percentage. It has really helped us. It has also helped to motivate workers. When you are motivated, it will improve your performance”. (Interview: HCW Buea)*

“*the PBF has really helped our community as we are able to fetch out paupers in the community and also get contracts which we did not have before” (FGD*: *CHW*, *Tiko)*

Community health workers also reported the importance of home visits but had a contrary view of how they were treated during them. They believed that without them, the nurses would be unable to conduct the home visits effectively as required by the PBF indicator because it is the CHWs who understand the community and the quarters, and providers acknowledged that without the CHWs, they would be unable to effectively implement the home visit. However, some CHWs expressed dissatisfaction regarding how they were paid for the home visits.

*“Last year home visit we were not being paid*. *We were paid only when we refer people and after that when they called*, *I just told them*, *I can’t be taking a nurse for a home visit she is the only one that is being paid and me taking her to the houses is not being paid*. *I refuse working for the home visit but with this new contract they have agreed to pay me*, *and I have decided to go for the home visit” (FGD: CHW, Tiko)*

The providers emphasized the importance of home visits and they have observed how these visits have helped to increase access to and utilization of maternal services in their communities. The providers reported that this initiative has had positive effects on access, as they are better able to relate to patients, locate drop out cases in the community, find poor and vulnerable individuals unable to access health facilities, nurses are able to visit patient and the community at home during home visits, and receive feedback from the community to support quality improvement of the health facilities.

“*Before PBF*, *there were no home visits and*, *we only went out when the Ministry organized campaigns such as*, *children and action or mother and childbirth week*. *It was held twice a year from April to June*, *for the first one and then November to December for the second one*. *The program entails going to the community and doing activities such as vaccinating the children with polio*, *vitamin A and deworm the children as well*. *This was from the instruction of the Ministry*, *because when they give instructions*, *they also provide funds*, *hence there was no autonomy*, *but with PBF*, *the hospital was given autonomy*, *and it is also an opportunity for the Community Health Workers to be engaged*. *In this facility*, *we have about 18 communities*, *and we target a community per month during which we do free consultations*, *free lab tests*, *and even malaria treatment and HIV test*, *we also counsel women who want to do family planning and administer it to them and those who also want to start antenatal care*. *So*, *it’s really good because with PBF*, *there is money to do all these things unlike before where there will be complaints of no money*. *It is another way of advertising our services to the community*, *more often*, *some don’t know about the existence of a facility like ours*. *It really benefits the community more for it helps the people*. *(Interview*, *HCW*, *Limbe*,*Buea)*

### Delay in payments

Delay in PBF payments was a dominant theme across all levels. Delays affected the implementation process at all levels, including the management of poor and vulnerable patients, especially in the private sectors. The providers’ primary challenge was the irregular payments of subsidies, which made it difficult for them to carry out their activities because they did not know when they would be receiving payments and—in some cases—they did not have the funds to cover the activities themselves. They reported that this delay in the payment of incentives influences recruitment and retention of staff and affect the effective implementation of the expectations for the poor and vulnerable as well as their performance.

Delay also created challenges at the level of supervision, as it left supervisors unable to provide effective coaching. The inability to provide effective coaching was not because of the escalating violence at the time of the project, but because of staff demotivation, as the facilities were continually asking for money. However, PBF seemed to have improved the resilience and motivation of the health facilities in these regions, as the providers were aware of the policy’s motivations (and knew that they would be paid someday, despite the delay), and there was an expectation that they would provide services during emergencies (e.g., internally displaced persons benefit under the category of “poor and vulnerable”). The providers’ indicated that delays in payment had had a significant impact on the implementation process. However, despite the identified challenges, PBF was overall perceived as an innovative approach that was helping to revamp the centralized budget design of the health system in Cameroon.

In view of the delay in payment and other challenges reported by the health providers and administrators, we probed further to understand if they would prefer the old system to PBF. All providers responded that they would prefer the PBF system to the old system.

*“If you were the one, what will you say, obviously we will prefer the PBF system to the old system” (Interview: HCW, Buea)*

Based on information available at the national and district level in Cameroon, PBF has helped to improve some aspects of the health budget, with substantial funds now directed toward the operational level (districts), thereby increasing autonomy and empowering health facilities. However, these positive changes were accompanied by serious challenges, which are discussed in the following paragraphs.

### Implementation challenges

In addition to the payment delays discussed above, the primary implementation challenges reported by participants were around quality, drugs, lack of documentation, verification, management of the poor and the vulnerable, and some contextual issues and the nature of private facilities.

*1. Meeting Minimum Standards for Quality of Care*: Participants expressed concern with the way they were assessed in relation to the minimum quality standards. Health facilities had different quality levels when they enrolled into PBF. Some health facilities had a very low-quality level, and it was very difficult for them to upgrade to the expected minimum within a short period. For example, some facilities became operational with limited staff, and consequently had poor quality adherence. If a health facility were offering consultations to 20 patients a month, when they enrolled in PBF, the expectation would be for this facility to expand the monthly offer of consultation to 50–100 patients. Therefore, it was challenging for them to progress. Although some of these facilities were working to meet the quality standards, attaining these remained a challenge.
*2. Management of Change*: Some participants noted that the change process remained a significant challenge. This was reported primarily by some experienced nurses, who saw the project as unfeasible and were thus unwilling to meet expectations and sought to undermine coaching sessions. They were not comfortable with new graduates—those with qualifications but limited experience—taking over supervision and focusing on quality standards, especially in the maternity department. Some nurses were resistant to change, but those who saw the advantages of PBF—the social entrepreneurs—reported that they were benefiting from embracing change. There were also health facilities that had used PBF money to construct full health centers, with recruits, and these were growing—whereas others remained unwilling to change their status quo.
*3. Verification Process*: The verification process considers a predefined established list of criteria to validate a consultation. These criteria include, for example, socio demographic characteristics of the patient must be complete—patient’s name, sex, age, and address. However, most providers did not routinely collect these data completely and the change process made it challenging for some. The verification agent requires these details to verify that this patient was the individual who visited the health facility. Patients are randomly selected from the register by the verification agent and their details are obtain from the register to locate the patient in the field to assess customer satisfaction and feedback from the patient. If they are unable to locate the patient at the specified address—or if the patient has not used the services in relation to the same visit as documented—this becomes an issue. This can create conflict between the verification agent and the health care provider.

The verification agent will review the facility registers to ensure that what is declared aligns with what is being verified. However, if there is any inconsistency in the reporting of up to 10% (known as error margin) for the specific indicator, the health facility is likely to lose the PBF incentive for that indicator for the reporting period. This is challenging for some health facilities, which have lost substantial sums of money because of this error margin. This error margin can be because of, but not limited to, a counting error, poor data entry for example, 100 consultations being counted as 80, or 100 registered as 120, and this has significant implications. It also results in a loss of data regarding the true workload, leaving poor quality data, and the verification process is costly. As some attempt to cheat the system, a rigorous verification mechanism was put in place. Some providers expressed concerns about the verification process, as they felt it might be affecting the quality scoring because they do not appreciate the random selection of patients to evaluate patient satisfaction on their quality as they believed some of the patients can be biased in their reporting.

In addition, before PBF, some facilities had only one register. They registered the number of beds, consultations, children, men, and rate of hospitalization, with everything documented in one register. With PBF, a register is needed for each activity, and some facilities find this obligation burdensome.

*4*. *Language translation*: Health providers reported challenges with the quality assessment checklist which was translated from the French language into English—and there were incorrect translations. This was related to some of the equipment, and health providers were penalized for over two years until a reviewers identified that the issue was related to the way the indicator was translated rather than that the health facility lacked the equipment specified in the checklist. This point was equally raised by CHWs during the FGDs.
*5*. *Business Plans and Indices Tools*: Business plans must be up to date, adhering to the regulations of the PBF expectations, with a minimum quality assessment score of above 50% if the contract is to be renewed. The rules are particularly strict for facilities that have had PBF for some time, with newer facilities usually given some time to meet quality expectations. It is not sufficient for a health facility to simply bring a business plan and sign a contract. The presentation of the indices tool and the business plan make the health facility more transparent, and some facilities find this challenging because it exposes certain details that were previously kept private. If the health facilities are unable to provide their indices tools, this suggests that they are hiding something from the public and, as a result, there will be no contract. Some of the health facilities reported that there were delays in receiving feedback from the PBF team on their business plans, and without the business plans, they could not proceed in the implementation of activities for the following quarters. Another great challenge is the development of the business plan; respondents suggested that the preparation of plans quarterly is not effective, and that it would be better to prepare semiannual plans given the delay in reviewing the plan and associated expectations.
*6*. *Escalating Conflict*: Some verification agents have been attacked during their verification processes, with reports destroyed and some agents even threatened and held against their will for several hours. This general atmosphere has made verification very difficult. Even going into the field for coaching has become very challenging. Some health facilities have been burnt down and some closed. With the escalating conflict, the equity scores were adjusted to reflect the need and situation of the region. Internally displaced persons can get access to services under the poor and vulnerable category.

### What facilitates the implementation process as reported by respondents?

#### Decentralization process

**1.** *Autonomy*: One participant reported that, “*the inception of PBF was grounded in autonomy*, *which is at the center of decentralization*. *Therefore*, *power is devolved to the lowest level—that is*, *autonomy—thus PBF serves decentralization*.*”* This decentralization allows the health facilities to be autonomous, which facilitates their work. This was reported primarily in the context of recruitment, participation and decision making, including the right to recruit staff without going through the regional delegate. Facilities can use these protocols, or they can enlist the help of the district medical officer, if necessary, to ensure authentication of certificates. This was reported at the health facilities as one of the major benefits of the decentralization.

**2.** *Equipment*: The health facilities used funds generated locally to acquire equipment. They no longer needed to write to central administration to request a microscope and then wait for a year or two for the equipment to arrive. In the past, when equipment was broken, it was necessary to send a letter to central administration to arrange repairs; and before the new equipment arrived, a patient could get worse, or the health facility might become frustrated and choose to stop offering certain services. As reported by one respondent,

“*There is a ministerial text giving the facility full authority to use funds generated locally to buy equipment and to renovate their buildings without bottlenecks in bureaucracy*, *as well as to other lap reagents*. *That is the decentralization already involved in the process”(Interview*: *Stakeholder)*.

Respondents reported that the only domain still facing problems is that of the management of drugs. The health facilities do not have the authority to buy drugs, sell them, and plough the profits back into their facilities where they are needed.

**3**. *Community health workers and home visits*: PBF has provided avenues for contracts with CHWs that were not previously available. CHWs work directly with the community, and their role is critical in care delivery. PBF contracts motivate CHWs in their work because they provide indicators to be met, putting them in an active position and creating financial motivation. In addition, women and the wider community can make their voices heard and share their expectations, using a feedback mechanism that promotes quality improvement. For example, women can press for the facilities to include family-planning components in their business plans, insisting that they are incorporated into indicators for CHWs and health facilities. In this way, women are empowered. Underserved populations are identified and given a voice, and their needs can be more easily addressed when they are incorporated into business plans and developed into indicators.

*4*. *Quality standards*: There is increased competition amongst health facilities in improving quality standards to stimulate demand. Some health facility administrators are using this as a mechanism to caution against provider’s poor communication attitudes towards patients.

“*Before now*, *we did not have so many private facilities around as at now*, *so we can have about an average of 25 birth deliveries per month but there are some months that it could reach up to sixty*. *So*, *with the stiff competition now*, *I think I can say our average deliveries has reduced*. *But they are a lot of things we are doing now to stay on top*. *I cannot really say if it’s because of the PBF program that the competition has increased because I do not know but we have what we are doing to keep up with these private facilities*. *We caution the mid wives whenever there is a complaint against them that they risk losing their PBF motivation if they do not change their attitudes*, *another complaint was about the limited private ward*, *so we are now working on that” (Interview*,*HCW*: *public facility*, *Buea)*.

Finally, looking back before PBF, the providers’ considered that there had been some remarkable changes in the way the system and their delivery of care. The health providers were very pleased that someone had come to interview them and to inquire about the challenges that they are facing since the introduction of the PBF policy, as they believed that their voices needed to be heard to inform policy implementation challenges.

## Discussion

The findings of this study revealed that PBF has played a strong role in the decentralization of the health sector and its budget to some extent. Based on providers’ experiences, PBF has improved on the quality of care, motivated providers, and helped with resource allocation. It also has the potential to reach the poor and vulnerable [14]. However, delays in payment are an important factor that affects the smooth implementation of PBF [14]. This has been reported in other countries and has a tremendous impact on the PBF policy in general and potential for sustainability [14,26,27]. The delays reported by providers were very demotivating and likely to affect the care provided to the poor and vulnerable [14].

Drawing from the PA perspective, there were experiences of tension in the transitioning process between the principal and the agent (providers) as reported by administrators and this tension is still ongoing with bureaucratic bottleneck at the central level affecting the smooth implementation of some of the PBF practices. Various levels of PA relationships as noted by Renmans et al. [1] can be observed, including hospital administrator/health providers, verification/health facility, health facility/patient, and health facility/CHW [1]. There are several actors involved in PBF and these are interlinked in PA theory [1] as seen in the CHW/provider relationship.

In a PA relationship, it is assumed that the health providers (agents) are better informed and giving them autonomy will increase their creativity and improve their strategies in achieving their predefined targets [1]. Some facilities have improved their quality of care and increased the funds they raise. These facilities see PBF as an “icing on the cake”. Some of these health facilities have taken advantage of PBF, which makes them social entrepreneurs. They see the benefits of embracing change and have used PBF incentives to construct and expand their health centers with recruits, whereas other facilities still face challenges regardless of what is provided.

Financial incentive is one of the most important components that requires careful consideration because it forms the basis of the PA theory [1]. Payment delays are an issue affecting specific functions in the implementation of PBF and the equity elements [14]. When payments are made promptly, health facilities are motivated in their supervision and coaching sessions. Verification agents can verify data on time and send reports in promptly to initiate payments and ensure that payments are made on time. However, the extent of decentralization is limited, and this, from a PA analysis, has implications for the implementation [1]. Financial incentive is central to the PA theory, and delays or unfairness in the distribution of incentives can jeopardize the PBF program in Cameroon [1,26].

The situation between the CHW and the health administrators can be reflected in the PA theory on information asymmetry where the CHW understand the community better than the nurses and health teams and they are the ones that will lead the nurses to get access to the households; however, they are not satisfied with what they get and, in some instances, they refused to adhere to the task until their request was considered. Also, potential conflict between the verifying agent and the health facility were reported.

### Sustainability considerations

Respondents perceived the introduction of PBF in the context of universal health coverage. They believe the government is making efforts and they see PBF as one of the front liners towards achieving universal health coverage, evaluating the level of preparedness of health facilities to embrace universal health coverage in terms of quality of health care, production, good managerial skills, and financial management. The Ministry of Public Health has established policies stating that PBF should be implemented in all health facilities (private, public, and confessional), without discrimination. The regulatory body is responsible for the accreditation of facilities after successful renewals of contracts over a period of three trimesters. This accreditation informs the public that the quality of health care offered by this facility is good. Therefore, facilities that work hard to improve their quality receive more clients and more money. Facilities that do not meet the quality standards will be penalized after coaching and re-coaching, and they will be given the option of restarting the PBF as a pilot study. A small number of health facilities have been through this process. Some were advised to benchmark with other facilities that were performing well to gain an understanding of how they could improve their performance. Facilities that are unable to meet the quality score can undergo coaching. If this failure persists for a long period, it may lead to some regulatory sanctions.

Providers reported that the sustainability of PBF greatly depends on political will, and they perceived it not from limitation of budget, but the will to fully incorporate the policy at all levels. The first step is the reallocation of the budget to ensure a reasonable amount is allocated to the operational level. This was perceived as an important policy change to support sustainability. The respondents reported that the government is making gradual changes to the input reform budget, introducing the PBF budget to ensure the sustainability of the system toward achieving universal health coverage. This process was piloted in 2017 in the Littoral and East regions, in link with the phasing of the implementation. There are still issues with the structures in place, in part because of corruption and in part because some regions do not seem to have the capacity to meet expectations and may require some time to establish the necessary structures. Some regions have experienced constant delays in payment which the respondents perceived as an issue within the public financial management system at the central level and administrative bottlenecks. Therefore, though PBF is a national policy, the actors at the central level, i.e., the Ministry of Public Health, are not perceived by agents to play their role effectively in enabling full implementation of PBF best practices and theories. Providers believed that regulators should be given their full autonomy and reduce administrative and/or financial bottle necks which tend to distort the smooth implementation of activities.

### Strengths and limitations

This study was limited to the Southwest region experiences of health care providers including focus group discussions with CHW. Though the timing of the interviews may have introduced potential recall bias, this was minimized with the use of multiple approaches in data collection, multiple interviews across health sectors and various level of health care providers and administrators, and two enrolment time points observed and compared across different districts and health facilities. Therefore, the responses provide useful information about the previous structures and previous experiences and the changes in relation to improved motivation and performance. As such, the findings may not reflect experiences in other regions, but provides an overview of the implementation challenges and perspectives of providers who have engaged with both the old system and the PBF system, which is strength as this is the first study to examine this aspect. The conflict may have been a limitation in relation to accessing some health facilities, but this did not have any effect on their reported experiences before and after PBF. Finally, this study is focused on overall provider’s experiences and did not look into any differences in providers’ perspectives on the PBF design phases from pilot to scale. The study did not target only the health facilities that were engaged in the pilot phase, but rather, providers that met our eligibility criteria at any of the eligible health facilities.

## Conclusion

PBF is a national strategy for achieving universal health coverage in Cameroon, and the experiences of providers provide a vital guide for national policy. The introduction of PBF has resulted in positive changes to quality of care when compared to the previous system. Addressing the delays in PBF payments will help to overcome the challenges to implementation and provide opportunities for health facilities to be more efficient and better improve on their performance.

## Abbreviations

PA: Principal Agent
FGD: Focus group discussion
COREQ: Criteria for qualitative interviews
PHC: Primary health care
MaxQDA: Qualitative data analysis software
KII: Key informant interviews
CHW: Community health worker
PBF: Performance-based financing
HCW: Health care worker
